# The Foreign Journals: Medicine

**Published:** 1839-04

**Authors:** 


### MEDICINE.

### *Camphor, a Panacea.* By M. RASPAIL.

IF physicians will only fairly try these new modes of smoking and sneezing out diseases, M. Raspail assures us that—we shall be astonished.

Let every man (says he) get a double-bottomed box, one part of which is to contain powdered camphor, the other camphor cigars, and thus he may walk through this dirty world without being defiled; and if he be defiled already, he will promptly be made clean. If a believer in camphor only take the precaution to place a lump of it on the pit of his stomach, the powder of it in his ears, the snuff of it in his nose, and the smoke of it in his mouth, he may walk through the hospitals with pleasure, he may investigate the plague in security, and instead of imbibing the miasma of infection, he will diffuse the odour of health. M. Raspail assures us that he has smoked camphor now for three months, and that whenever he leaves it off he becomes sensible of some inconvenience. M. Raspail does *not* inform us how many suffer no inconvenience who have smoked no camphor. But M. Raspail presses for a fair trial; we give, therefore, a brief description of his apparatus, and add the names of those maladies which he has successfully treated.

1st. The camphor made into impalpable powder forms the snuff.

2d. The cigars are made by stuffing pieces of straw, or crow-quills, with small lumps of camphor, and stopping the ends with (papier joseph) blotting paper. These cigars, however, are to be smoked *cold*, i. e. the air is to be sucked through them impregnated by the camphor; and the saliva is to be swallowed.

3d. Pieces of linen soaked in a saturated solution of camphor in alcohol, and covered by Indian-rubber cloths, or other skins, or bladders, made impervious to the air by gum or starch. (p. 12.)

But M. Raspail becomes serious; he prays that "MM. les Médecins" would believe that he has not exaggerated the effect of his apparatus; and that he has witnessed a host of evils exorcised by them as by magic,—obstinate diseases, resisting every other treatment. "I beseech them, therefore," says M. R. "to try for themselves. I appeal, not to their memories, but to their consciences; and the conscience *of the physiologist* is based entirely upon experiment."

M. R. mentions coughs, "rhumes," catarrhs, "grippe," oppression, expectoration, hooping-cough, and croup, as yielding to the camphor cigar and snuff. He has no doubt, too, of its salutary effect upon the first stage of phthisis pulmonalis; also, the affections of the stomach which have not yielded to antiphlogistic remedies. In diseases of the abdomen, the clothes soaked with the saturated solution are "heroic,"—also in intermittent fevers, enteritis, cholera, and typhoid and yellow fevers; in diseases of the liver, the spleen, the kidneys, the uterus, &c. Finally, camphor has proved successful in curing diseases of the ears and eyes; in removing cutaneous disorders, and arresting the agonies of tooth-ache.

*Gazette des Hôpitaux, Nov.* 17, 1838.

*On the Character and Origin of the Pustula Maligna.* By Dr. SCHWABE.

THE pustula maligna appears at first as a small, dark, reddish spot, which is shortly transformed into a small vesicle containing a whitish yellow fluid. The vesicle is at first surrounded by a red shining areola, which soon becomes of a darker colour, and the surrounding cellular tissue becomes indurated. The vesicle now increases in size, and is sometimes as large as a hazel nut; the swelling of the surrounding parts at the same time becomes greater, and the adjacent glands begin to get swollen and painful. Generally about the second or third day, but sometimes within a few hours, the pustule becomes at first of a blue and then of a black colour, and the areola takes on a livid tint. As soon as the pustule breaks, its contents, spreading in all directions, infect the healthy skin, and give rise to new pustules. The skin covering the pustule mortifies, and a black slough is formed, which extends rapidly in all directions, and is tolerably firmly connected with the neighbouring healthy parts. The surrounding tumour is now doughy, and the skin immediately around the pustule is hard and leathery. The gangrene affects merely the skin and the subjacent cellular and adipose tissues; the muscles, nerves, and blood-vessels remain healthy.

At first there are no general symptoms; the patient feels a sharp pricking pain, which he ascribes to the sting of an insect, and pays no attention to it. It is only after the areola begins to turn livid, and the surrounding tumour doughy, that the system is attacked. This occurs sometimes in a few hours, sometimes in two or three days. The patient becomes feverish, the pulse is hard and quick; the respiration difficult; there is great prostration of strength, nausea, sleeplessness, and delirium. The pulse soon becomes small, frequent, and intermitting; and is at last scarcely perceptible. The delirium becomes constant, and there is great restlessness and utter prostration. The tongue and lips are covered with dry dark crusts; the fæces are dark coloured and very offensive, and death ensues in from four to seven days from the commencement of the affection, with all the symptoms of a putrid typhus.

Dr. Schwabe believes that the disease is produced by the contagion of glanders, applied in most instances by insects. He has observed, that the patients in general complained of a momentary prick, as if by the sting of an insect, on the spot where the pustule afterwards appeared; that they were in the open air at the time, and that the pustules never appeared where the body was protected by the dress. In six cases, too, which occurred to him, none of the patients had come in contact with diseased cattle, nor had any of them partaken of newly-slaughtered flesh for several days previous.

Dr. S. has never seen the disease propagated from one individual to another, although, in two cases which came under his notice, the children slept with the affected parent.

The treatment which Dr. S. recommends, is the local application of muriatic acid till a line of demarcation forms, and the gangrenous mass be thrown off.

*Casper's Wochenshrift. Nov.* 13, 1838.

---

*On the Treatment of Quinsy by Scarification.* By M. GÉRARDIN.

M. VELPEAU, in his "Traité d'Anatomie Chirurgicale," distinguishes two species of inflammation in the tonsils, one limited to the mucous membrane, the other situated in the submucous cellular tissue. It is important to recognize these two different seats of the disease. The examination of the throat will be sufficient to determine whether the mucous tissue or the cellular be attacked; and this diagnosis is indispensable, since the treatment which may prove successful in the one case would be detrimental in the other. Perhaps it is to the want of this distinction, that the experience of one practitioner has been contradicted by another: for instance, in the use of alum gargles; which have proved advantageous in the mucous inflammations, but have increased the pain and inflammatory symptoms in the parenchymatous disease.

The application of leeches to the submaxillary region occasions often a local subcutaneous effusion in the neighbourhood of the inflamed tonsils; and it is to be lamented that this remedy should still be the common routine prescription with the generality of practitioners; for the physician is often called in only after one or two applications of leeches have preceded him. In every quinsy, whether mucous or parenchymatous, if the state of the subject be plethoric, I first bleed from the arm, and some minutes afterwards I proceed to scarify the inflamed parts. It is rarely necessary to recur to this operation more than twice. In the most intense parenchymatous quinsies the disease yields to two scarifications made within twelve or twenty-four hours of each other. The swelling subsides directly, the patient feels great relief, which he does not fail to express. The scarifications are to be made more or less deep, according to the seat of the inflammation; if the cynanche is mucous, I slightly scarify all the parts reddened by the inflammation, the tonsils, the palate, and the uvula: if the disease is parenchymatous, I make deeper scarifications, particularly in the tonsils. I puncture the surface as long as the flowing blood will permit me to see; and, after it has cleared away, as the operation is not painful, I complete the scarifications on the untouched parts. In the parenchymatous quinsy, twelve or fifteen punctures will afford a sufficient bleeding. Under the influence of the scarifications, the resolution of the inflammation is prompt and invariable, and takes place almost always the day after the operation. After some time, there will be observed small white lines—the cicatrices of the punctures. I know of no objections to this practice, but the difficulty of getting at the seat of the disease, or when the intensity of the inflammation prevents the opening of the jaws. Boyer speaks strongly of scarifications in cynanche tonsillaris, but only as an occasional operation. It is remarkable that he should not have recommended them more generally, after using these words: "by scarification the alarming progress of the symptoms is arrested, and a prompt relief is given to the state of anxiety under which the patient labours." I scarify at the commencement of the inflammation, or at its height, according to the time of my arrival.

I have also recourse to scarifications in laryngitis and pharyngitis, and always with the greatest success. They are certainly more efficacious than cupping, and avoid the marks in the neck.

*Bulletin de l'Academie Royale de Médecine.* Tom. cxi. No. 1. 1838.

---

*Case of Fatal Inflammation of the Vermiform Process.* By Dr. BIESKE.

L. H. aged twenty, tall, but of robust constitution, and previously in the enjoyment of good health, complained of being sick and uncomfortable on the evening of the 2d September. In the early part of the day he had been in excellent spirits, and had taken a hearty dinner. On the 3d he complained of want of appetite, weariness, pains in the limbs, and a slight pain in a circumscribed spot, of about three inches in diameter, in the right iliac region, which was somewhat increased by pressure. The tongue was coated, and the pulse rather frequent. The disease was considered as a slight febrile attack, eight leeches were applied to the painful part, and a dose of acetate of potash given internally. In the evening the pain was removed, and the patient appeared to be doing well. An emulsion of castor oil with laurel water was given to open the bowels. The condition of the patient remained much the same during the 4th; on the 5th he was more restless, and complained of a return of the pain in the side, which was again removed by the application of leeches. In the evening there were still no unfavorable symptoms, but on the morning of the 6th matters had assumed a different aspect; the pulse could scarcely be counted, was small, hard, and wiry, the abdomen tense and tympanitic, but not painful; the face collapsed, and the extremities cold. The patient was immediately bled, but his weakness prevented more than two cups of blood being taken. In spite of a variety of remedies, his state went on from bad to worse, and he expired at one o'clock on the morning of the 7th.

On dissection, the processus vermiformis was found in a state of mortification, and a concretion, about the size of a large coffee berry, the probable cause of the disease, was found impacted in it. A section of the concretion showed its nucleus to be formed by the stone of a grape.

*Rust's Magazin für die gesammte Heilkunde.* Vol. lii. Part 2.

---

*On the Local Employment of Mercury in the Treatment of Variolous Eruptions.* By M. BRIQUET, Physician to the Hôpital Cochin.

THE chief object of this paper is to point out the great advantages derived from the application of mercurial plasters (Emplâtre de Vigo) in the treatment of all the varieties of small-pox, whether simple, confluent, or modified. The effect of these plasters is, in general, either to prevent the development of the pustules, or so to modify them that they become mere abortions, and very slightly affect the skin. M. Briquet details several cases which support him in his opinion that mercury, locally applied, exercises great influence on the course and nature of the eruption. By the application of the plasters the exanthema either undergoes complete resolution, or it is converted into vesicles or tubercles. The resolution is either primitive or secondary. The former takes place when the plaster has been applied whilst the eruption is still papular, and its effect is the complete disappearance of a number of papulæ, which, without the application of the plaster, would have passed into pustules. This diminution of number, as ascertained by counting the number of papulæ before and after the application of the plaster, varies from a third to a tenth of the whole number. Secondary resolution ensues, when the papulæ, after being covered by the plaster, increase in size for two days, and then pass into resolution. But the most general effect of the plasters is to produce conversion of the eruption into a vesicular or tubercular form. The vesicular is the more common, and in such cases the eruption bears considerable resemblance to herpes, or to vesicles which have been developed under a cataplasm. The vesicles, when they have reached their maximum development, contain a milky fluid; their walls are formed by an epidermis extremely thin, and not at all tense, and their base is sometimes surrounded by a slight areola of a pale rose colour. They vary in size; the smallest are not larger than the point of a pin, the largest equal a millet-seed. The skin between the individual vesicles is pale and white, and never red and swollen as in pustular small-pox.

The slightest friction destroys the epidermis of the vesicles, and their base then appears as a moist, slightly red surface, which, on the day after the removal of the plaster, has already become dry and covered by a delicate epidermis. No scales are ever formed on this surface. In no case has M. Briquet ever seen a permanent cicatrix formed, except in one which was treated by M. Nonat, and in which there were a number of very minute cicatrices, but still widely different from the deep marks of common small-pox. In this case M. Briquet doubts whether the plaster had been rightly applied.

In order that the plasters may have the effect of modifying the eruption, they must be applied before it has become pustular; when applied later than the fifth day, they do not appear to exert any beneficial influence.

The conversion into tubercles is more rare, and generally takes place in cases of confluent small-pox. They harden and desquamate, soon after removal of the plaster, without leaving any permanent cicatrix.

The plaster is best applied spread upon some coarse stuff, stiff enough to support itself, and thus remain in exact contact with the skin. A little mercurial ointment is applied to the eyelids and nostrils, as the plaster cannot readily be kept upon these parts. The plaster is allowed to remain for three days in simple small-pox, and a day longer in confluent cases. No benefit is derived from a longer application, but rather the reverse, as softening of the base of the vesicles may ensue in consequence, and cicatrices be formed.

When the plasters are allowed to remain too long, a slight erysipelas may be the consequence, but this is extremely rare. In two cases an eruption resembling measles followed the application of the plasters, but it did not appear certain that it was in consequence of them. A slight eczema is sometimes produced by the plasters, but its extent is always very limited, and it is of little consequence. In no case was any deleterious effect produced by thus modifying the eruption, but the advantages appear to be many. The inflammation that would otherwise have ensued in confluent cases was obviated, and the brain thus in all probability prevented from being affected.

The mercury seems to be the chief agent in effecting the modification. Pressure did not produce the same effects, for the pustules were developed as fully below adhesive plaster as upon the free surface of the skin. Neither have the plasters of lead any effect in changing the nature of the eruption; but the modification is produced by mercurial ointment spread upon the surface, equally well, if not better than by the mercurial plaster.

*Archives Générales de Médecine. Octobre*, 1838.

---

### *On the Origin, Growth, and Termination of Pulmonary Tubercle.* By Professor A. A. SEBASTIAN.

IF pulmonary tubercles were, as some writers maintain, formed in the cellular tissue of the organ, they would, without doubt, most frequently be found in that separating the lobules and lobes, yet there I have never been able to discover them. Hence, though not perfectly convinced of the fact, I am inclined to place the seat of tubercle in the pulmonary cells. The process of injecting the bronchi throws no light on the question, for though I never detected the mercury I had injected by those tubes in the tubercles, the fact of its not having entered them may be accounted for either by the cells being completely stuffed with tubercular matter, or by the pressure exercised on the cells by other tubercles, possibly formed in the cellular membrane. The smallest tubercles I have seen were certainly larger than the pulmonary cells, the diameter of which, according to Weber, equals from 0·053 to 0·16 of a Paris line. With respect to the connexion of tubercle and hydatid in the lower animals, Kuhn has shown that, besides the cyst proper to the hydatid and the external cyst, consisting of plastic lymph, it is produced through the irritation o. the foreign body. Once formed this external cyst secretes a yellow, soft substance, full of calcareous principles. In proportion as it increases in quantity, this yellow or tuberculous substance presses the acephalocyst to such an extent, that in the end a mere vestige of it is only to be found imbedded in the yellow matter. It does not appear probable that human tubercles originate in an hydatid, for those of the lungs and exterior surface of the peritoneum are not invested by a cyst. If such were the mode of formation of that product in men, hydatids would be met with frequently in conjunction with it; now, I have never by the closest scrutiny succeeded in discovering cysts and tubercles together. Again, with respect to the primary form of the morbid deposit under consideration, Professor Carswell does not appear to have proved that the formation of the semi-transparent substance really precedes that of acknowledged tubercle. The production of that matter in the instances described might have been the result of irritation caused by the presence of tubercles in neighbouring parts. So, as regards the peritoneum, in his theory, any given tubercle might have existed before the plastic lymph thrown out through the irritation of other and neighbouring tubercles. And this mode of viewing the question acquires greater weight from the observation of the Professor himself, that the semi-transparent matter does not always exist. Tubercle, in its very earliest stage, always appeared to me in the form of a minute white cloud placed in healthy parenchyma and imperfectly circumscribed, at least not round. Possibly this cloudy appearance is cadaveric, and the matter has been pellucid during life; in this case in order to become a tubercle it must have acquired opacity before death. I have besides frequently observed points growing from these minute clouds and ascertained that in process of time they enlarge, or rather that several of them coalesce and by their union form the *miliary tubercles* of Laennec; these last I therefore believe to be true tubercles. According to Schroeder Van Der Kolk and Dr. Carswell, the form of tubercles depends on the structure of the lung. I conceive, however, that it is in some measure influenced by the nature of the tuberculous substance itself. For in proportion as the tubercle increases in size, the cellular membrane gives way before it, and hence it is that the form of larger tubercles is nearly the same in every organ. I do not understand how the resistance offered by the tissue of organs can, as Dr. Carswell supposes, increase the hardness of tubercles. Murdoch states that those occasionally met with in the medullary substance of the brain are harder than those of other organs. According to Dr. Alexander Thompson,* tubercles are not destitute of organization and really contain vessels. This uncommon notion appears to me to be explicable by the fact that tuberculous matter is sometimes deposited round a small artery or vein, so that upon injection of the pulmonary vessels a branch appears in the midst of the tubercle. But no ramusculi can be detected given off from that branch to the tubercle itself, and the large size of the branch, too, is enough to show that it could not belong to that body. I have in my possession several fragments of lung in which a small tubercle is seen in the midst of well injected pulmonary tissue in such manner that the red material used for the injection of the artery, the blue for that of the vein, and the white for that of the bronchi, immediately invest the tubercle. Whence, I conclude, that no argument can be drawn from the condition of the parts surrounding the smallest tubercles in support of their inflammatory origin. I do not, indeed, affirm that the adjacent tissue is healthy, but I contend that it presents no manifest and visible signs of any morbid state. Again, I have never seen hepatization immediately around minute tubercles. Nor can I omit, what appears to me of the last importance in this matter, namely, that tubercles cease to be formed in the substance of the lungs when once it has commenced to exhibit manifest signs of inflammation round those bodies in the crude state. *Tijdschrift v. N. Gesched.* ii. d. 3 st.

* London Medical and Surgical Journal; December, 1833.

